# InterLINCing Chromatin Organization and Mechanobiology in Laminopathies

**DOI:** 10.1007/s11886-023-01853-2

**Published:** 2023-04-13

**Authors:** Parisha P. Shah, Garrett T. Santini, Kaitlyn M. Shen, Rajan Jain

**Affiliations:** 1 Departments of Medicine and Cell and Developmental Biology, Penn Cardiovascular Institute, Penn Epigenetics Institute, Perelman School of Medicine, University of Pennsylvania, Philadelphia, PA 19104 USA; 2 Smilow Center for Translational Research, 09-184, 3400 Civic Center Blvd., Philadelphia, PA 19104 USA; 3 Smilow Center for Translational Research, 09-101, 3400 Civic Center Blvd., Philadelphia, PA 19104 USA

**Keywords:** Lamin, Laminopathy, Epigenetics, Mechanobiology

## Abstract

**Purpose of review:**

In this review, we explore the chromatin-related consequences of laminopathy-linked mutations through the lens of mechanotransduction.

**Recent findings:**

Multiple studies have highlighted the role of the nuclear lamina in maintaining the integrity of the nucleus. The lamina also has a critical role in 3D genome organization. Mutations in lamina proteins associated with various laminopathies result in the loss of organization of DNA at the nuclear periphery. However, it remains unclear if or how these two aspects of lamin function are connected. Recent data suggests that unlinking the cytoskeleton from the nuclear lamina may be beneficial to slow progress of deleterious phenotypes observed in laminopathies.

**Summary:**

In this review, we highlight emerging data that suggest interlinked chromatin- and mechanical biology-related pathways are interconnected in the pathogenesis of laminopathies.

## Introduction: The Nucleus Organizes Chromatin

The nucleus is a complex organelle that encloses, organizes, and regulates multiple aspects of the genome. Metazoan nuclei feature a meshwork of proteins at the inner nuclear membrane surface termed the “nuclear lamina” [1]. The nuclear lamina provides integrity to the nucleus and acts as a scaffold for chromatin organization [1–5]. Pathogenic mutations and haploinsufficiency in genes encoding nuclear lamina proteins, particularly *LMNA*, result in a collection of syndromes referred to as laminopathies, which includes myopathies, lipodystrophies, neuropathies, and segmental progeroid syndromes [6]. Laminopathies are associated with loss of chromatin organization [7–10, 11•, 12], compromised signal transduction [13–16], and aberrant mechanical transduction (hereafter mechanotransduction) [17–19]. In this review, we examine the chromatin-related consequences of laminopathy-linked mutations through the lens of mechanotransduction, highlighting emerging data that suggest interlinked chromatin- and mechanical biology-related pathways are at the nexus of laminopathy pathogenesis.

Within the context of laminopathies, we will first discuss the normal role of the nuclear lamina in chromatin organization before exploring the impact of mechanical forces on lamina-mediated organization. The nuclear lamina is critical for normal epigenetic gene regulation and genome organization [1, 20, 21]. Chromatin is radially organized within the nucleus, with some chromatin regions positioned at the nuclear periphery versus other regions more centrally positioned within the nucleus. A subset of peripheral chromatin physically contacts the nuclear lamina, in regions termed lamina-associated domains (LADs) [22]. In studies using various forms of genomic mapping, LADs comprise ~ 30–40% of the total genome and are highly gene depleted [22–24]. Moreover, genes localized within LADs are typically transcriptionally silenced and enriched for hallmarks of heterochromatin, including di- and trimethylation of lysine 9 on histone H3 (H3K9me2 and H3K9me3) [22, 25, 26]. Therefore, it is unsurprising that pathogenic or haploinsufficient *LMNA* mutations, which are linked to laminopathies, have adverse effects on the epigenome [27]. In brief, across various models, pathogenic *LMNA* mutations or reductions have been linked to aberrant changes in higher order chromatin organization, LAD positioning, DNA methylation, distribution of post-translational histone modifications, heterochromatin localization, and transcription. We refer readers to other recent reviews for additional details [7, 23, 28]. While the specific mechanisms underlying these defects remain the subject of study, it is generally appreciated that reductions or defects in nuclear lamins are linked to a weakened nuclear membrane [29, 30], which itself is a critical factor in responding to mechanical stress and cues [19], as discussed next.

## Nuclear Organization and Mechanotransduction: Mechanical Signals are Sensed by the Nucleus and Impact Genome Organization

Cells and tissues are continually exposed to stress from various sources. Mechanical stress can result in alterations to the structure and function of cells, including rapid transcriptional responses, in a process referred to as mechanosensing [31]. Mechanically-induced stimuli are transduced by structural elements of the cellular membrane, cytoplasm, and nuclear envelope, as well as mechanosensing nuclear ligands and signal transduction factors in both the cytoplasm and nucleus, ultimately converging on transcriptional responses. Extensive studies have explored the impact of mechanical stress on cells and are well-reviewed [17, 31, 32], and we highlight key studies supporting the hypothesis that the nucleus can directly or indirectly respond to extracellular forces. First, the Ingber group established that external force impacts normal nuclear shape. The team demonstrated that micropipette indentation on the surface of endothelial cells resulted in significant nuclear deformation [33]. Other foundational work from the Discher laboratory revealed that the stiffness of the nuclear lamina matrix, via concentration changes of nuclear lamins, scales with the amount of mechanical stress in specific tissue types [34]. These data strongly indicate that nuclei can both sense and respond to differing amounts of physiological mechanical stress. Reciprocally, in an ex vivo cartilage study, enzymatic degradation of factors contributing to extracellular forces resulted in a reduction of nuclear membrane stiffness [35], again suggesting a functional link between mechanical force and nuclear response.

In considering the role of the nuclear membrane in chromatin organization, several studies provide specific support for chromatin changes in response to mechanical force changes. In a custom-culture neuronal cell model, repeated high impulse mechanical loading resulted in increased dynamic nuclear response, deformations in nuclei, and chromatin displacement [36]. Similarly, mesenchymal stem cells grown in a tunable hydrogel system responded to increased extracellular matrix stiffness by increasing histone acetylation (via downregulation of histone deacetylase) and nuclear stiffness, which led to a specific osteogenic fate commitment [37]. In another hydrogel model, porcine aortic myofibroblasts that underwent persistent activation from stiff hydrogels were unable to deactivate, even after the stimulus was removed. Moreover, following persistent activation, myofibroblasts developed condensed chromatin and reduced chromatin accessibility, attributed to decreased histone acetylation by upregulation of histone deacetylase [38]. These and other studies strongly indicate a role for the nuclear lamina in maintaining normal genome integrity and organization in response to extracellular mechanical stress.

## Defects in Nuclear Lamins may Impact Mechanosensing

It is paramount to understand how normal levels of mechanical force or stress are impacted in laminopathies, where defects in normal nuclear lamina components weaken the nucleus, affect genome integrity, and are consequently associated with chromatin organization changes. It is noteworthy that cells with pathogenic laminopathy mutations or reductions in normal lamina components result in defective nuclear mechanosensing and exciting to address how this defect may be linked to disease pathogenesis. To this end, nuclear viscoelasticity is decreased in lamin A/C knockout mouse embryonic fibroblasts (using magnetic nanorods) [39]. Similarly, our group has shown reductions in viscoelasticity in human induced pluripotent stem cell (hiPSC) derived cardiac myocytes harboring pathogenic *LMNA* mutations [11•]. Nevertheless, it remains unanswered if cytoplasm-nuclear force communication plays a role in these phenotypes and if mutations in other components of mechanosensing machinery result in similar pathogenic phenotypes.

The studies highlighted above showcase the impact of manipulating extracellular force on nuclei, but it is important to consider how *normal* mechanical load and extracellular forces are linked to the nucleus. In higher eukaryotes, force propagated through the cytoskeleton is “communicated” to the nucleus by the linker of nucleoskeleton and cytoskeleton (LINC) complex, a protein network comprised of emerin (*EMD*), SUN (*SUN1* and *SUN2*) and nesprin (*SYNE1* and *SYNE2*) proteins [40, 41]. The LINC complex physically connects to the nuclear lamina via SUN domain proteins and to the cytoskeleton via the KASH domain of the nesprin family proteins [42]. Directly relevant to this review, several groups have demonstrated an epistatic relationship between the LINC complex and lamin A/C in various models, as outlined below.

First, in a murine model, laminopathy phenotypes and reduced lifespan observed upon global *Lmna* loss were significantly ameliorated with global deletion of *Sun1* [43]. More recently, the Stewart laboratory utilized a conditional *Lmna* knockout murine model to expand this observation. They confirmed that organism-wide *Lmna* deletion in a *Sun1*^-/-^ background doubled the lifespan of the mouse and further showed that cardiac-specific *Lmna* deletion in a *Sun1*^*-/-*^ background ameliorated gross nuclear defects and organizational changes in cardiomyocytes, significantly reducing cardiac pathology [44••]. The authors then employed a dominant-negative SUN1 protein that uncoupled SUN-nesprin interactions. Expression of the dominant-negative SUN1 protein in *Lmna* deficient mice phenocopied *Sun1* deletion, suggesting that lamin A/C deficiency requires the LINC complex-associated role of SUN1 or that prevention of the lamin A/C deficiency defects requires the concomitant loss of the LINC complex-associated role of SUN1. Of note, the longevity of the *Sun1*^*-/-*^ mice is surprisingly relatively preserved and is indicative of considerable functional, cell type-specific, or combinatorial redundancy in force sensing factors. These studies highlight the interplay between force propagating factors and the nuclear lamina components collectively suggesting that normal mechanical force is detrimental to cells with compromised nuclear lamina integrity, which can be at least partially relieved by eliminating force sensing in the impaired nuclei.

In another recent study, researchers have shown cardiac myocytes integrate mechanical cues during differentiation, concordant with chromatin changes [45••]. Specifically, the team showed that intranuclear mechanical tension spatially correlates with H3K9me3-marked chromatin. Changes in nuclear deformation, either through disruption of LINC complex proteins or manipulation of normal environmental stiffness led to changes in the pattern of H3K9me3-marked chromatin by immunofluorescence. This could be due to multiple possible mechanisms, including the dissociation of H3K9me3-marked chromatin from the nuclear periphery, the redistribution of H3K9me3-marked chromatin, or the de novo formation of heterochromatin. Distinguishing these different possibilities will be critical to understand the molecular basis for how mechanobiology impacts genome organization. The authors provided additional support for observations that chromatin organization can be abrogated by manipulating stiffness. Their work is consistent with an emerging theme that dynamic mechanical environmental cues have a functional role in cardiac differentiation [46, 47] and development, perhaps through changes to nuclear architecture.

Further underscoring this connectedness, the Goldman laboratory has demonstrated that A- and B-type lamins interact with different components of the cytoskeleton via the LINC complex to regulate various mechanical pathways in mouse embryonic fibroblasts (MEFs) [48]. Specifically, A-type lamins (lamins A and C) interact with F-actin and vimentin intermediate filaments (VIFs) to modulate cortical stiffness, cytoplasmic stiffness, and cell contractility. In contrast, the B-type lamins (lamins B1 and B2) mainly interact with VIFs to modulate cytoplasmic stiffness and cell contractility. The team then used dominant negative LINC constructs and RNA interference to specifically ablate regions of the LINC complex that interact with F-actin and VIFs, which resulted in decreased cortical stiffness or cytoplasmic stiffness, similar mechanical phenotypes to cells that lack A- or B-type lamins, respectively. These results provide additional evidence for the nuclear lamina and LINC complex working in concert to modulate the mechanical properties of a cell.

Given the compelling functional link between mechanical force and nuclear architecture, it is critical to understand what happens to a *normal* nucleus in the absence of *normal* force sensing. Intriguingly, it has been observed that patients with mutated LINC components, including mutations in the nesprin proteins SYNE1 and SYNE2*,* phenocopy laminopathies and can develop muscular dystrophy, dilated cardiomyopathy (DCM), and some aspects of neuropathy [49]. A mouse *Syne1* knockout model (nesprin-1 lacking the SUN protein-interacting KASH domain) showed decreased survival, attenuated growth, and aberrant nuclear positioning in skeletal muscle [50]. The authors demonstrated defects in transmission of normal force and strain in muscle fibers lacking nesprin-1, collectively suggesting that nesprin-1 is essential for normal genome organization and nuclear positioning in skeletal muscle. Similarly, targeted deletion of *Syne1* and *Syne2* in murine cardiac myocytes resulted in early onset cardiomyopathy and cells with aberrant nuclear positioning, shape, and chromatin organization [51]. In this study, deletion of either *Syne* or both ablated gene expression changes in response to mechanical stimuli, again linking normal mechanical stimulation to functional gene expression responses. The McNally laboratory generated a mouse model where the C-terminus of nesprin-1 was deleted, ablating all SUN-nesprin-1 protein interactions [52]. Homozygous mutant mice exhibited significant lethality and surviving mice had severe limb muscle and cardiac defects. Additionally, the Prosser laboratory has demonstrated that desmin, a muscle-specific intermediate filament, is required to maintain normal gross nuclear morphology and LAD-mediated genome organization via interactions with LINC complex proteins in rat cardiac myocytes [53].

## Aberrant Chromatin Organization may Link Mechanosensing- and Lamina-Mediated Laminopathy Disease Progression

The chromatin organization defects in pathogenic *LMNA* mutants, the link between mechanosensing proteins and the nuclear lamina, the role of normal mechanical cues to functional chromatin organization changes, the overlap of phenotypes in pathogenic *LMNA* and *SYNE* mutations, and the ability to ameliorate a murine laminopathy by the deletion of a force sensing LINC protein collectively raise the interesting possibility that mechanical and lamina/chromatin-mediated mechanisms are linked in the manifestation of laminopathies. This connection is starting to be more closely examined, including work by the Wickstrom laboratory, which has determined that chromatin can alter its own mechanical state to maintain genome integrity in response to nuclear deformation [54••]. Specifically, the team showed that cells counteract mechanical stretch that deforms the nucleus by reducing H3K9me3-marked heterochromatin. They also show that persistent stretch can result in adaptation of cells within tissues to redistribute mechanical force, allowing cells to restore normal chromatin states. Relatedly, the Mauck and Lakadamyali laboratories showed that changes in the substrate stiffness of tenocytes and mesenchymal stromal cells were linked to changes in chromatin compaction, which were reversible in healthy cells by dynamically stiffening the substrate [55].

Repeated mechanical stress and nuclear rupture causes DNA damage [56]. Laminopathy models have suggested that *LMNA* mutations weaken the nuclear lamina, causing both increased nuclear rupture and DNA damage that likely contribute to disease pathology [57, 58•]. Notably, the specific laminopathy Hutchinson-Gilford Progeria Syndrome, is a disease of accelerated aging and prominently features a high degree of DNA damage [59], and the link between laminopathies and DNA damage is an exciting and well-reviewed area research [60]. Of interest to the scope of this review, both lamins A/C and LINC complex components promote DNA damage repair [61–63]. Further, the Lammerding laboratory recently showed mechanical trauma of *Lmna* mutant myocytes caused nuclear envelope rupture and DNA damage [58•]. When the research team disrupted the LINC complex via dominant negative KASH, this ameliorated rupture and improved contractility. They further showed that muscle biopsies taken from patients with *LMNA* muscular dystrophy also demonstrated an increased DNA damage phenotype. Taken together, these results emphasize a role for mechanically-induced DNA damage in laminopathy pathophysiology and disease progress, opening the exciting possibility that such a pathway could be considered for therapeutic targeting to improve disease outcomes, should DNA damage be an important contributor to laminopathy phenotypes [64].

**Fig. 1:**
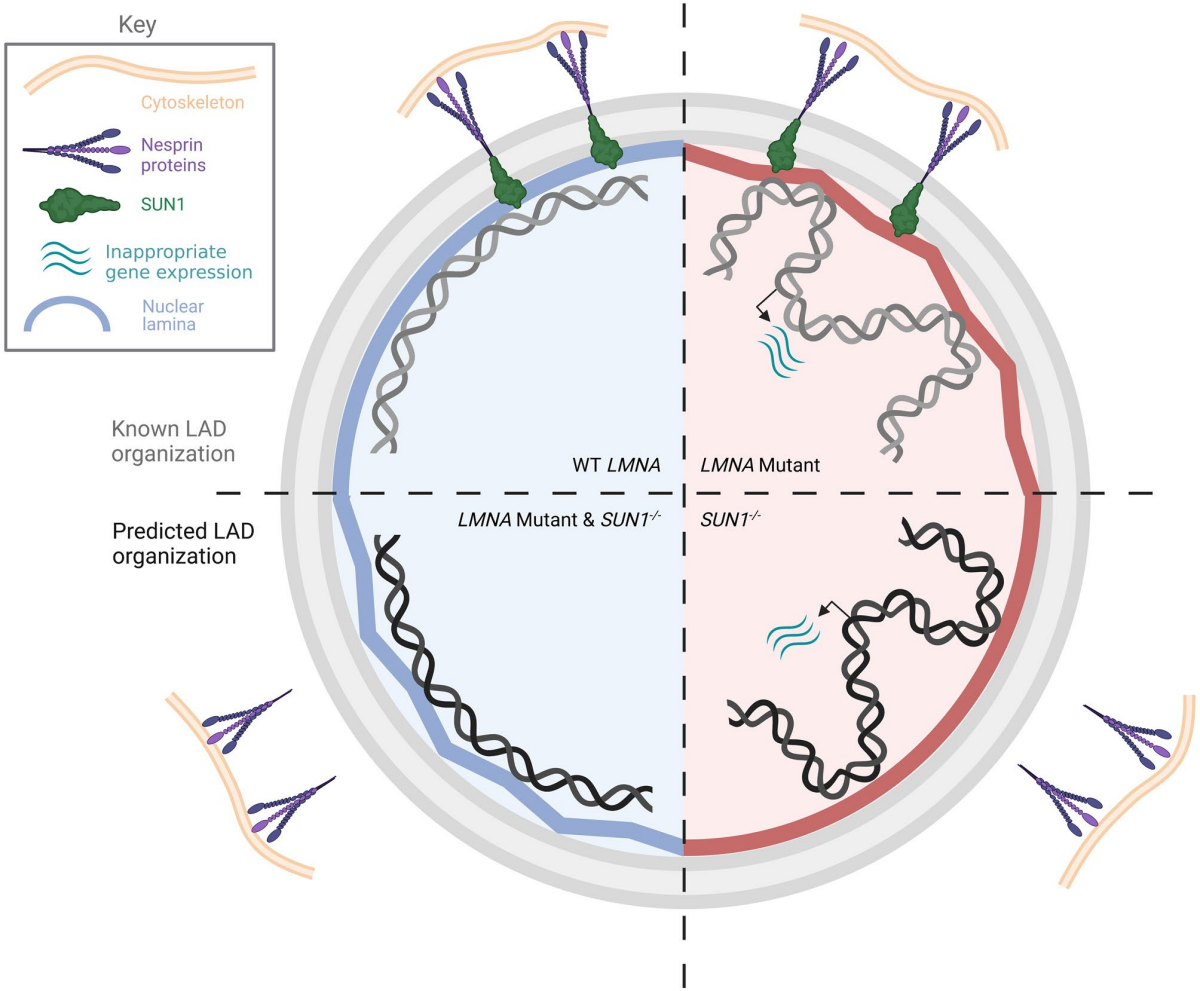
Model: Mechanical force is critical for normal genome organization. Top left: Wild type cells exhibit normal force transduction to the nucleus via the LINC complex and have normal LADs at the nuclear periphery. Top right: Cells with perturbations of the nuclear lamina via mutations in *LMNA* feature compromised LADs and aberrant upregulation of non-lineage genes. Bottom right: Cells with perturbations to the LINC complex via *SUN1* deletion *may* demonstrate compromised LAD organization, leading to aberrant gene expression and failure to properly respond to mechanical stimuli. Bottom left: Perturbation of the LINC complex in *LMNA* mutant conditions *may* preserve LAD organization and prevent abnormal gene expression, based on observations of *Lmna* mutant defects (partially) ameliorated with concomitant *SUN1* loss. Figure created with BioRender.com

Collectively, these studies strongly support the idea that a function shared by the LINC complex and lamin proteins is affected by mechanical stress. One possible model is that mechanical force, as sensed by LINC complex components, is critical for normal genome organization through the connection to an intact nuclear lamina (Fig. 1). In pathogenic *LMNA* mutant cells or in cells with reduced *LMNA* levels, the normal lamina integrity is compromised, resulting in gross nuclear abnormalities, DNA damage, and loss of LADs [8, 11•]. Therefore, one can speculate that gross nuclear morphology and chromatin organization defects may be the result of normal mechanical force no longer being tolerated or properly sensed in cells with compromised nuclear lamina integrity (Fig. 1, top right). This model provides exciting opportunities to test aspects of the mechanosensing and lamina connection to better understand their interplay in laminopathy disease progression. First, as discussed above, loss of nesprin proteins results in some laminopathy-like phenotypes. This suggests that mechanosensing is also required to maintain normal genome organization and nuclear integrity, even in the presence of otherwise normal nuclear lamina components (Fig. 1, bottom right). However, this has not been empirically demonstrated and the molecular similarities and differences between mutations in nesprin versus lamin proteins also remain unknown. An initial step in understanding this connection is to map LADs in cells either lacking individual nesprin proteins, expressing the aforementioned dominant-negative peptides, or harboring patient-inspired mutations. If LADs are lost, are they similar to those LADs lost in pathogenic *LMNA* mutants? If so, it is compelling to focus future studies on understanding temporal dynamics of this dissociation and if similar gene expression changes are linked to this LAD dissociation.

Relatedly, as presented above, mechanical force that results in nuclear deformation results in release of peripheral heterochromatin to maintain nuclear integrity, shearing, or rupture [54••]. In *LMNA* mutants, it is possible that the normal amount of mechanical force upon a nucleus can no longer be tolerated because the lamina itself is fragile and mechanically strained [18] and no longer appropriately scaled with the normal amount of force [19, 34]. This can be tested by uncoupling the force sensing mechanism from the nuclear lamina. For example, in a cultured cell system, it is possible to express pathogenic *LMNA* mutants or deplete *LMNA* in cells where *SUN1* is also deleted, or to express the aforementioned dominant-negative peptides that uncouple nesprin-SUN interactions. If gross nuclear morphology and LAD organization are preserved, this would suggest that a nucleus with impaired strength or integrity manifests morphology and organization defects because it cannot properly withstand normal levels of mechanical stress or force (Fig. 1, bottom left). Of note, this experiment would be designed to preserve normal nuclear morphology and organization upon abrogating lamina integrity. It is much more challenging to consider a situation in which abnormal LAD organization would be *restored* after it was disrupted. To date, it remains elusive how LADs become established each cell cycle, and proteins that tether LADs to the nuclear periphery are also mostly unknown in mammalian cells [65, 66]. Moreover, cardiac cells are post-mitotic, and it is not yet clear if it is possible for LAD organization to change without a cell cycle. These and similar efforts underway by many research teams will continue to uncover additional critical factors, mechanisms, and pathways impacted in laminopathies, revealing the interlinked contributions of chromatin organization and mechanosensing in disease progression.

## Conclusion

The nuclear lamina plays multiple integral roles in the cell, including providing structural support for the nucleus and integrating cytoskeletal forces impinging on the LINC complex and nuclear envelope. In the nucleus, the lamina plays an important scaffold for chromatin to be organized in three-dimensional space. Emerging data suggests an intricate interplay between these various functions that may contribute to development and disease.

## References

[1] Dechat T (2008). Nuclear lamins: Major factors in the structural organization and function of the nucleus and chromatin. Genes Dev.

[2] Andres V, Gonzalez JM (2009). Role of A-type lamins in signaling, transcription, and chromatin organization. J Cell Biol.

[3] Burke B, Stewart CL (2013). The nuclear lamins: Flexibility in function. Nat Rev Mol Cell Biol.

[4] Poleshko A, et al. Genome-nuclear lamina interactions regulate cardiac stem cell lineage restriction. Cell. 2017;171(3):573–87 e14. 10.1016/j.cell.2017.09.018. 10.1016/j.cell.2017.09.018 PMC5683101 29033129

[5] Poleshko A, et al. H3K9me2 orchestrates inheritance of spatial positioning of peripheral heterochromatin through mitosis. Elife. 2019;8. 10.7554/eLife.49278. 10.7554/eLife.49278 PMC6795522 31573510

[6] Worman HJ, Bonne G (2007). Laminopathies: a wide spectrum of human diseases. Exp Cell Res.

[7] Briand N, Collas P (2018). Laminopathy-causing lamin A mutations reconfigure lamina-associated domains and local spatial chromatin conformation. Nucleus.

[8] Cheedipudi SM (2019). Genomic reorganization of lamin-associated domains in cardiac myocytes is associated with differential gene expression and DNA methylation in human dilated cardiomyopathy. Circ Res.

[9] Harr JC (2020). Loss of an H3K9me anchor rescues laminopathy-linked changes in nuclear organization and muscle function in an Emery-Dreifuss muscular dystrophy model. Genes Dev.

[10] Nikolova V (2004). Defects in nuclear structure and function promote dilated cardiomyopathy in lamin A/C-deficient mice. J Clin Invest.

[11] • Shah PP, et al. Pathogenic LMNA variants disrupt cardiac lamina-chromatin interactions and de-repress alternative fate genes. Cell Stem Cell. 2021;28(5):938–54 e9. 10.1016/j.stem.2020.12.016. **This study showed that LMNA mutations can cause lineage-specific changes in peripheral chromatin that correlate with inappropriate gene expression. It is possible this phenomenon occurs in LINC complex mutations and may be prevented in simultaneous LMNA and LINC perturbation**. 10.1016/j.stem.2020.12.016 PMC8106635 33529599

[12] Bertero A (2019). Chromatin compartment dynamics in a haploinsufficient model of cardiac laminopathy. J Cell Biol.

[13] Maraldi NM, Capanni C, Cenni V, Fini M, Lattanzi G (2011). Laminopathies and lamin-associated signaling pathways. J Cell Biochem.

[14] Muchir A (2007). Activation of MAPK pathways links LMNA mutations to cardiomyopathy in Emery-Dreifuss muscular dystrophy. J Clin Invest.

[15] Muchir A, Worman HJ (2016). Targeting mitogen-activated protein kinase signaling in mouse models of cardiomyopathy caused by lamin A/C gene mutations. Methods Enzymol.

[16] Wu W, Muchir A, Shan J, Bonne G, Worman HJ (2011). Mitogen-activated protein kinase inhibitors improve heart function and prevent fibrosis in cardiomyopathy caused by mutation in lamin A/C gene. Circulation.

[17] Donnaloja F, Carnevali F, Jacchetti E, Raimondi MT. Lamin A/C mechanotransduction in laminopathies. Cells. 2020;9(5). 10.3390/cells9051306. 10.3390/cells9051306 PMC7291067 32456328

[18] Lammerding J (2004). Lamin A/C deficiency causes defective nuclear mechanics and mechanotransduction. J Clin Invest.

[19] Swift J, Discher DE (2014). The nuclear lamina is mechano-responsive to ECM elasticity in mature tissue. J Cell Sci.

[20] Luperchio TR, Wong X, Reddy KL (2014). Genome regulation at the peripheral zone: Lamina associated domains in development and disease. Curr Opin Genet Dev.

[21] Shevelyov YY, Ulianov SV. The nuclear lamina as an organizer of chromosome architecture. Cells. 2019;8(2). 10.3390/cells8020136. 10.3390/cells8020136 PMC6406483 30744037

[22] Guelen L (2008). Domain organization of human chromosomes revealed by mapping of nuclear lamina interactions. Nature.

[23] van Steensel B, Belmont AS (2017). Lamina-associated domains: Links with chromosome architecture, heterochromatin, and gene repression. Cell.

[24] Shah PP, Keough KC, Gjoni K, Santini GT, Abdill RJ, Wickramasinghe NM, Dundes CE, Karnay A, Chen A, Salomon REA, Walsh PJ, Nguyen SC, Whalen S, Joyce EF, Loh KM, Dubois N, Pollard KS, Jain R. An atlas of lamina-associated chromatin across twelve human cell types reveals an intermediate chromatin subtype. Genome Biol. 2023 Jan 23;24(1):16. 10.1186/s13059-023-02849-5. PubMed PMID: 36691074; PubMed Central PMCID: PMC9869549. 10.1186/s13059-023-02849-5 PMC9869549 36691074

[25] Chen X, Yammine S, Shi C, Tark-Dame M, Gondor A, Ohlsson R (2014). The visualization of large organized chromatin domains enriched in the H3K9me2 mark within a single chromosome in a single cell. Epigenetics.

[26] Wen B, Wu H, Shinkai Y, Irizarry RA, Feinberg AP (2009). Large histone H3 lysine 9 dimethylated chromatin blocks distinguish differentiated from embryonic stem cells. Nat Genet.

[27] Malashicheva A, Perepelina K. Diversity of nuclear lamin A/C action as a key to tissue-specific regulation of cellular identity in health and disease. Front Cell Dev Biol. 2021;9:761469. 10.3389/fcell.2021.761469. 10.3389/fcell.2021.761469 PMC8548693 34722546

[28] Santini GT, Shah PP, Karnay A, Jain R (2022). Aberrant chromatin organization at the nexus of laminopathy disease pathways. Nucleus.

[29] Nmezi B (2019). Concentric organization of A- and B-type lamins predicts their distinct roles in the spatial organization and stability of the nuclear lamina. Proc Natl Acad Sci USA.

[30] Sullivan T (1999). Loss of A-type lamin expression compromises nuclear envelope integrity leading to muscular dystrophy. J Cell Biol.

[31] Davies PF, Tripathi SC (1993). Mechanical stress mechanisms and the cell. An endothelial paradigm. Circ Res.

[32] Martino F, Perestrelo AR, Vinarsky V, Pagliari S, Forte G (2018). Cellular mechanotransduction: From tension to function. Front Physiol.

[33] Maniotis AJ, Chen CS, Ingber DE (1997). Demonstration of mechanical connections between integrins, cytoskeletal filaments, and nucleoplasm that stabilize nuclear structure. Proc Natl Acad Sci USA.

[34] Swift J et al.. Nuclear lamin-A scales with tissue stiffness and enhances matrix-directed differentiation. Science. 2013;341(6149):1240104. 10.1126/science.1240104. 10.1126/science.1240104 PMC3976548 23990565

[35] McCreery KP, et al. Nuclear stiffness decreases with disruption of the extracellular matrix in living tissues. Small. 2021;17(6):e2006699. 10.1002/smll.202006699. 10.1002/smll.202006699 PMC7891867 33470544

[36] Schneider SE, et al. Dynamic biophysical responses of neuronal cell nuclei and cytoskeletal structure following high impulse loading. Acta Biomater. 2022. 10.1016/j.actbio.2022.07.002. 10.1016/j.actbio.2022.07.002 PMC10019187 35811070

[37] Olivares-Navarrete R, et al. Substrate stiffness controls osteoblastic and chondrocytic differentiation of mesenchymal stem cells without exogenous stimuli. PLoS One. 2017;12(1):e0170312. 10.1371/journal.pone.0170312. 10.1371/journal.pone.0170312 PMC5240960 28095466

[38] Walker CJ (2021). Nuclear mechanosensing drives chromatin remodelling in persistently activated fibroblasts. Nat Biomed Eng.

[39] Lee JS (2007). Nuclear lamin A/C deficiency induces defects in cell mechanics, polarization, and migration. Biophys J.

[40] Crisp M (2006). Coupling of the nucleus and cytoplasm: Role of the LINC complex. J Cell Biol.

[41] Lombardi ML, Lammerding J (2011). Keeping the LINC: The importance of nucleocytoskeletal coupling in intracellular force transmission and cellular function. Biochem Soc Trans.

[42] Kim DI, Birendra KC, Roux KJ (2015). Making the LINC: SUN and KASH protein interactions. Biol Chem.

[43] Chen CY (2012). Accumulation of the inner nuclear envelope protein Sun1 is pathogenic in progeric and dystrophic laminopathies. Cell.

[44] •• Chai RJ, et al. Disrupting the LINC complex by AAV mediated gene transduction prevents progression of Lamin induced cardiomyopathy. Nat Commun. 2021;12(1):4722. 10.1038/s41467-021-24849-4. **This study demonstrated that mutating or ablating the LINC complex increases lifespan of*****Lmna*****mutant mice, providing critical evidence for the interrelationship between the nuclear lamina and LINC complex and suggesting that targeting the LINC complex may be beneficial in treating laminopathies**. 10.1038/s41467-021-24849-4 PMC8342462 34354059

[45] •• Seelbinder B, et al. Nuclear deformation guides chromatin reorganization in cardiac development and disease. Nat Biomed Eng. 2021;5(12):1500–1516. 10.1038/s41551-021-00823-9. **In this study, the research team showed nuclear deformation of cardiac myocytes via environmental stiffening or LINC disruption abrogated chromatin reorganization and resulted in re-localization of H3K9me3-marked chromatin from the nuclear periphery. This study provided important evidence that mechanical cues are integrated into normal developmental pathways, stabilizing cell fate, via chromatin reorganization**. 10.1038/s41551-021-00823-9 PMC9300284 34857921

[46] Fukui H (2021). Bioelectric signaling and the control of cardiac cell identity in response to mechanical forces. Science.

[47] Jain R, Epstein JA (2021). Not all stress is bad for your heart. Science.

[48] Vahabikashi A, et al. Nuclear lamin isoforms differentially contribute to LINC complex-dependent nucleocytoskeletal coupling and whole-cell mechanics. Proc Natl Acad Sci USA. 2022;119(17):e2121816119. 10.1073/pnas.2121816119. 10.1073/pnas.2121816119 PMC9170021 35439057

[49] Mejat A, Misteli T. LINC complexes in health and disease. Nucleus. 2010;1(1):40–52. 10.4161/nucl.1.1.10530. 10.4161/nucl.1.1.10530 PMC3035119 21327104

[50] Zhang J (2010). Nesprin 1 is critical for nuclear positioning and anchorage. Hum Mol Genet.

[51] Banerjee I, et al. Targeted ablation of nesprin 1 and nesprin 2 from murine myocardium results in cardiomyopathy, altered nuclear morphology and inhibition of the biomechanical gene response. PLoS Genet. 2014;10(2):e1004114. 10.1371/journal.pgen.1004114. 10.1371/journal.pgen.1004114 PMC3930490 24586179

[52] Puckelwartz MJ (2009). Disruption of nesprin-1 produces an Emery Dreifuss muscular dystrophy-like phenotype in mice. Hum Mol Genet.

[53] Heffler J (2020). A balance between intermediate filaments and microtubules maintains nuclear architecture in the cardiomyocyte. Circ Res.

[54] •• Nava MM, et al. Heterochromatin-driven nuclear softening protects the genome against mechanical stress-induced damage. Cell. 2020;181(4):800–17 e22. 10.1016/j.cell.2020.03.052. **This study demonstrated two critical aspects of the impact of mechanical force on nuclei and genome organization: 1) that mechanical deformation of nuclei can be “counteracted” by release of heterochromatin from the nuclear periphery and 2) that failure for the nuclei to adapt to mechanical stress results in DNA damage. This important work links chromatin reorganization to the mechanical state of a cell in an adaptation model for maintaining genome integrity**. 10.1016/j.cell.2020.03.052 PMC7237863 32302590

[55] Heo SJ, Thakur S, Chen X, Loebel C, Xia B, McBeath R, Burdick JA, Shenoy VB, Mauck RL, Lakadamyali M. Aberrant chromatin reorganization in cells from diseased fibrous connective tissue in response to altered chemomechanical cues. Nat Biomed Eng. 2023 Feb;7(2):177–91. 10.1038/s41551-022-00910-5. Epub 2022 Aug 22. PMID: 35996026; PMCID: PMC10053755. 10.1038/s41551-022-00910-5 PMC10053755 35996026

[56] Shah P, et al. Nuclear deformation causes DNA damage by increasing replication stress. Curr Biol. 2021;31(4):753–65 e6. 10.1016/j.cub.2020.11.037. 10.1016/j.cub.2020.11.037 PMC7904640 33326770

[57] Broers JL, Kuijpers HJ, Ostlund C, Worman HJ, Endert J, Ramaekers FC (2005). Both lamin A and lamin C mutations cause lamina instability as well as loss of internal nuclear lamin organization. Exp Cell Res.

[58] • Earle AJ, et al. Mutant lamins cause nuclear envelope rupture and DNA damage in skeletal muscle cells. Nat Mater. 2020;19(4):464–73. 10.1038/s41563-019-0563-5. **This work provided evidence for two foundational topics in this review: 1) that*****LMNA*****mutations caused nuclear fragility and rupture and 2) that LINC complex perturbation ameliorated a*****LMNA*****mutant phenotype**. 10.1038/s41563-019-0563-5 PMC7102937 31844279

[59] Gonzalo S, Kreienkamp R (2015). DNA repair defects and genome instability in Hutchinson-Gilford Progeria Syndrome. Curr Opin Cell Biol.

[60] Gonzalo S (2014). DNA damage and lamins. Adv Exp Med Biol.

[61] Gonzalez-Suarez I, Redwood AB, Gonzalo S (2009). Loss of A-type lamins and genomic instability. Cell Cycle.

[62] Gonzalez-Suarez I (2009). Novel roles for A-type lamins in telomere biology and the DNA damage response pathway. EMBO J.

[63] Lawrence KS (2016). LINC complexes promote homologous recombination in part through inhibition of nonhomologous end joining. J Cell Biol.

[64] Kirby TJ, Zahr HC, Hannah Fong EH, Lammerding J. Eliminating elevated p53 signaling in Lmna-mutant mice fails to rescue skeletal muscle defects or extend survival. bioRxiv. 2022;2022.07.08.499329. 10.1101/2022.07.08.499329. 10.1038/s41420-024-01998-1 PMC11111808 38778055

[65] Gonzalez-Sandoval A (2015). Perinuclear anchoring of H3K9-Methylated chromatin stabilizes induced cell fate in C. elegans embryos. Cell.

[66] Kind J, van Steensel B (2010). Genome-nuclear lamina interactions and gene regulation. Curr Opin Cell Biol.

